# Supplementary figures and images for: RNA structural probing of guanine and uracil nucleotides in yeast
Source: PLoS One. 2023 Jul 7;18(7):e0288070. doi: 10.1371/journal.pone.0288070 (PMC10328344; doi:10.1371/journal.pone.0288070)

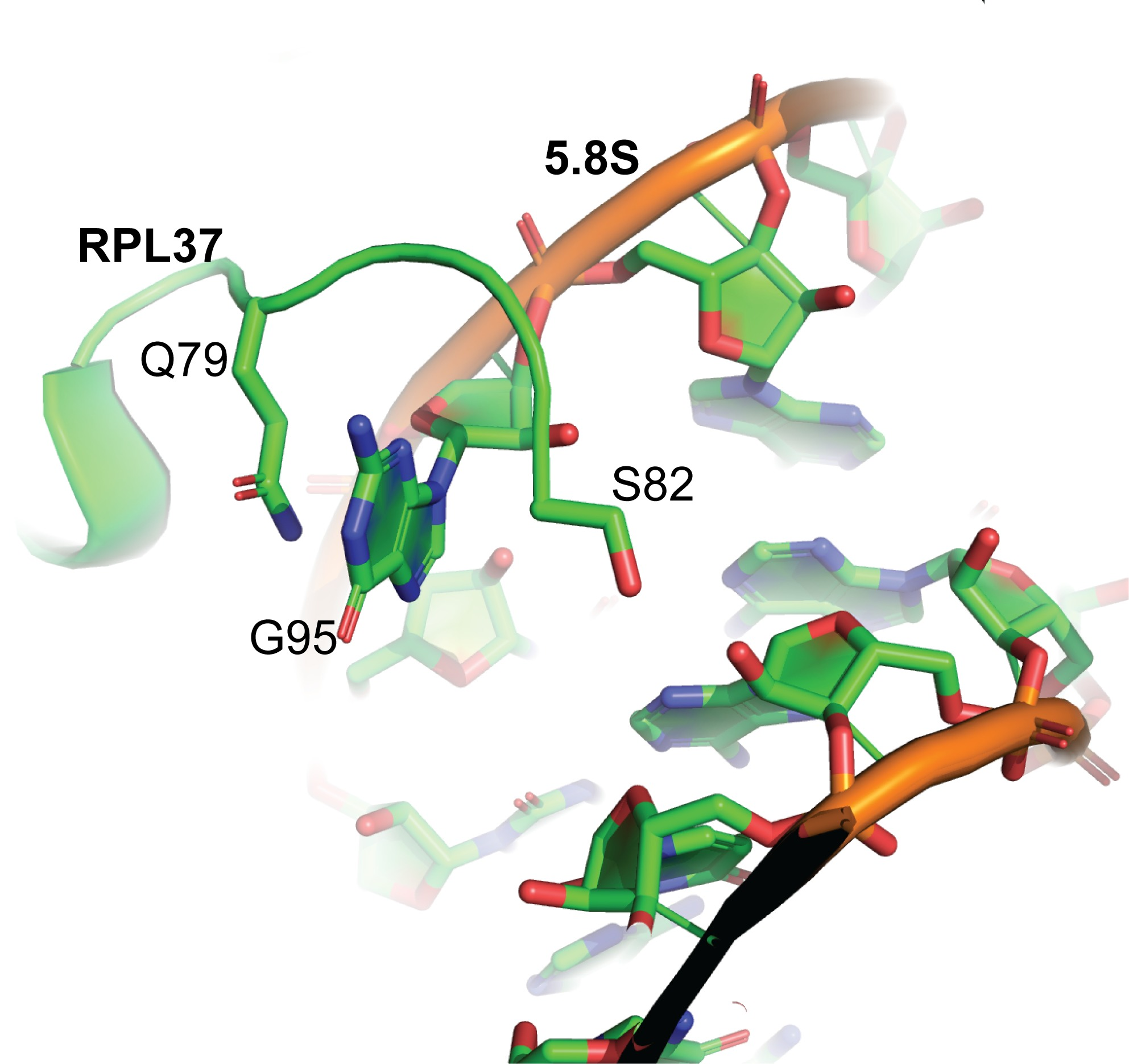

Supplement: S1 Fig — Position of 5.8S G95 and RPL37 Gln79 and Ser82 are marked (PDB id: 6TNU). Blue and reg colors represent nitrogen and oxygen, respectively. The phosphate backbone is in orange. (TIF) [file pone.0288070.s001.tif]
