# Supplementary material for: RNA structural probing of guanine and uracil nucleotides in yeast
Source: PLoS One. 2023 Jul 7;18(7):e0288070. doi: 10.1371/journal.pone.0288070 (PMC10328344; doi:10.1371/journal.pone.0288070)
Supplement: S1 Raw images — (PDF) [file pone.0288070.s002.pdf]

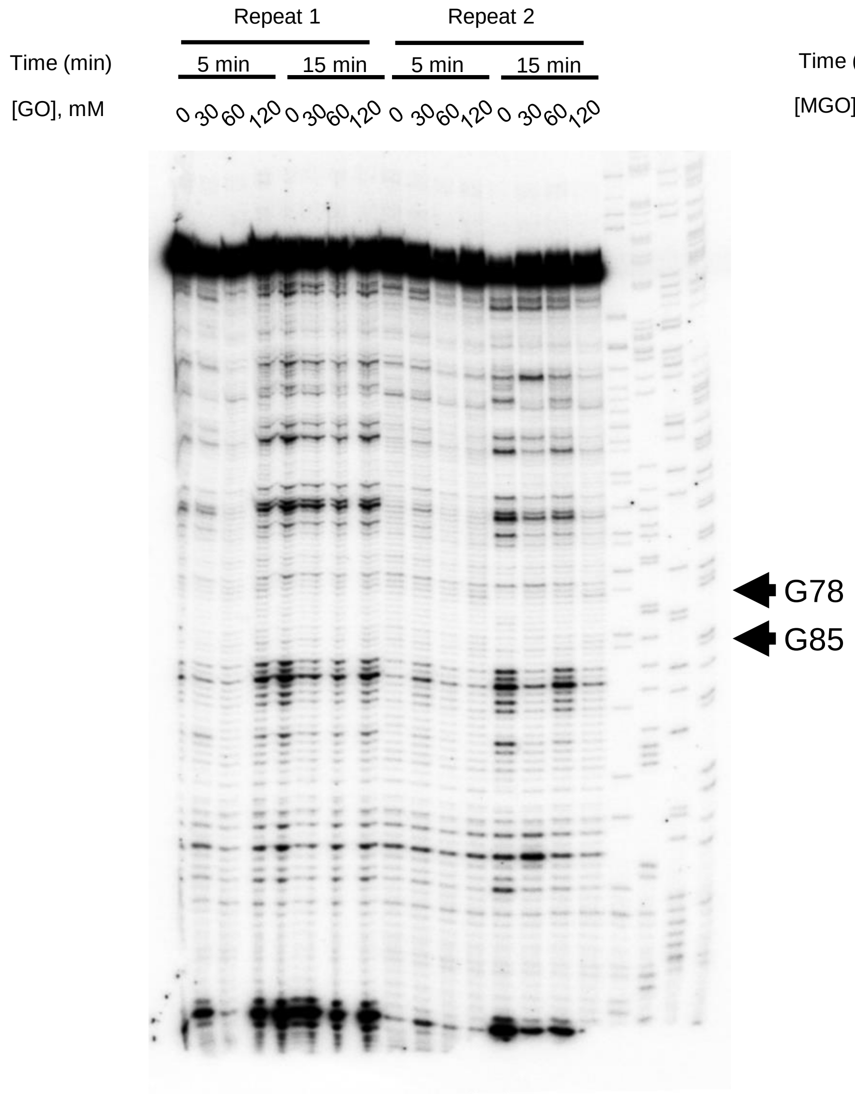

GO

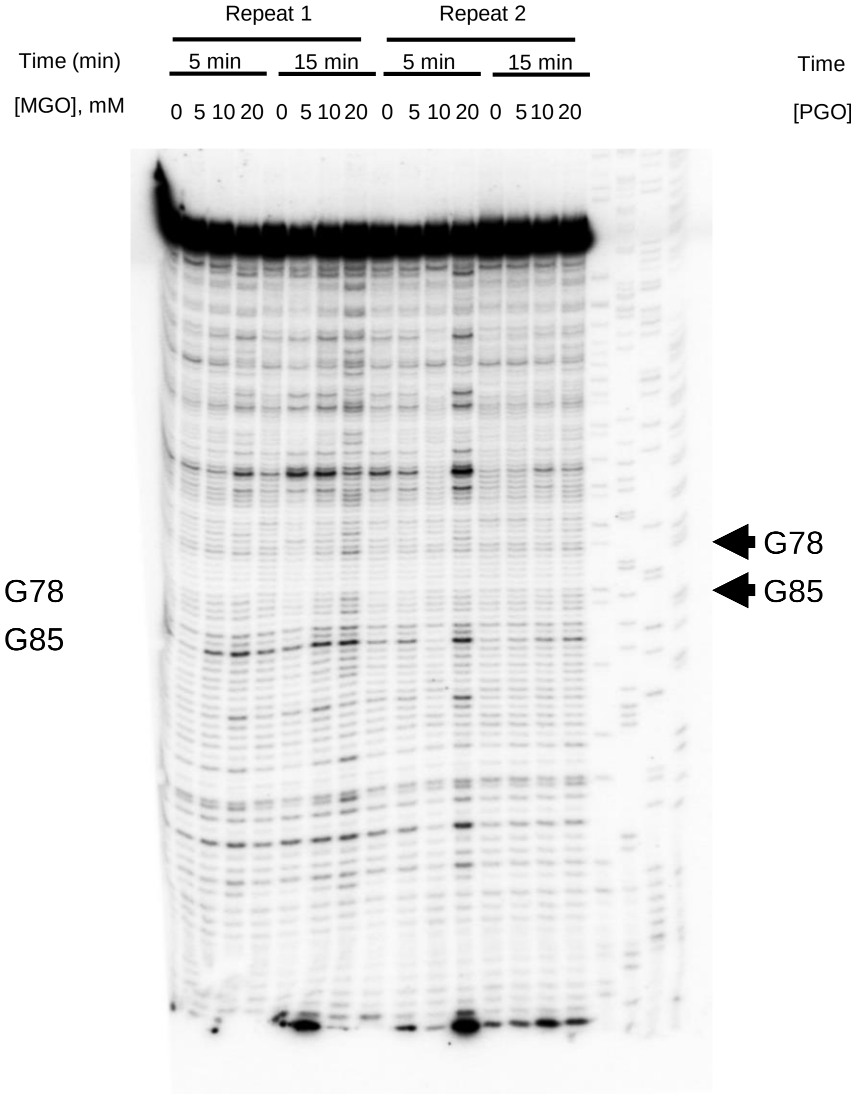

MGO

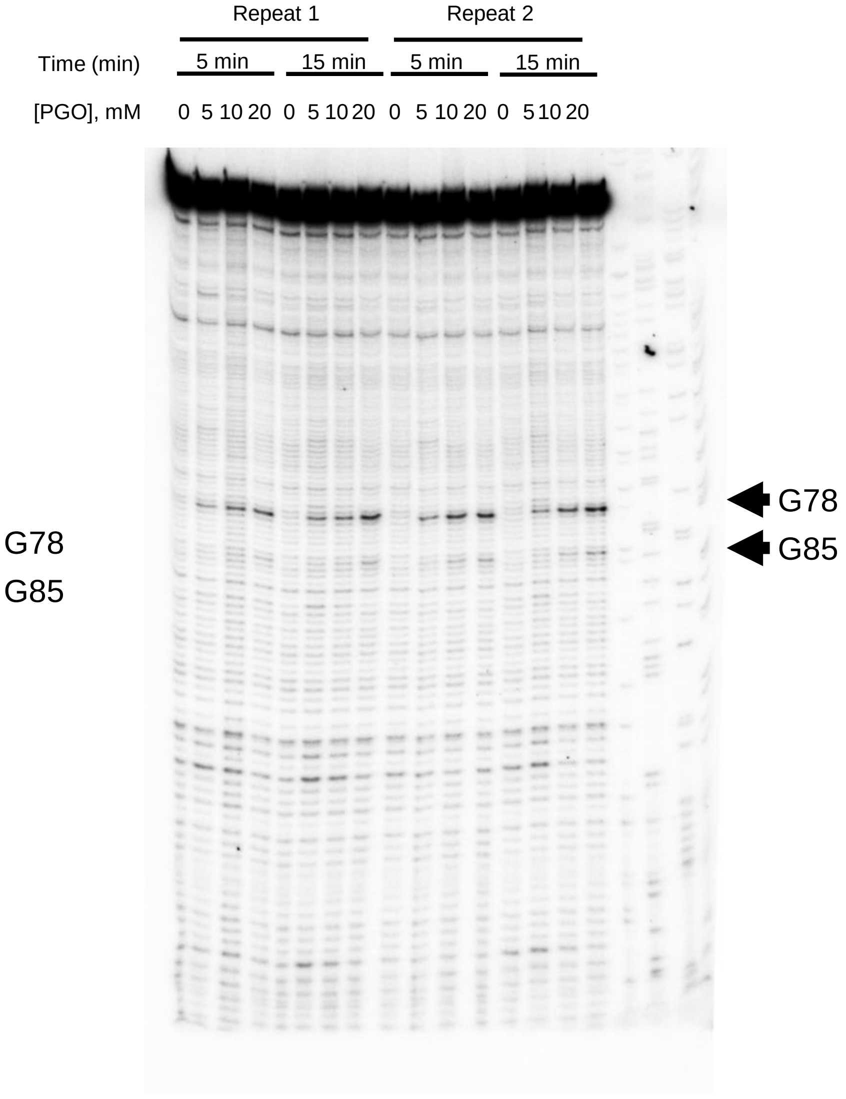

PGO

Original gel: Figure 1

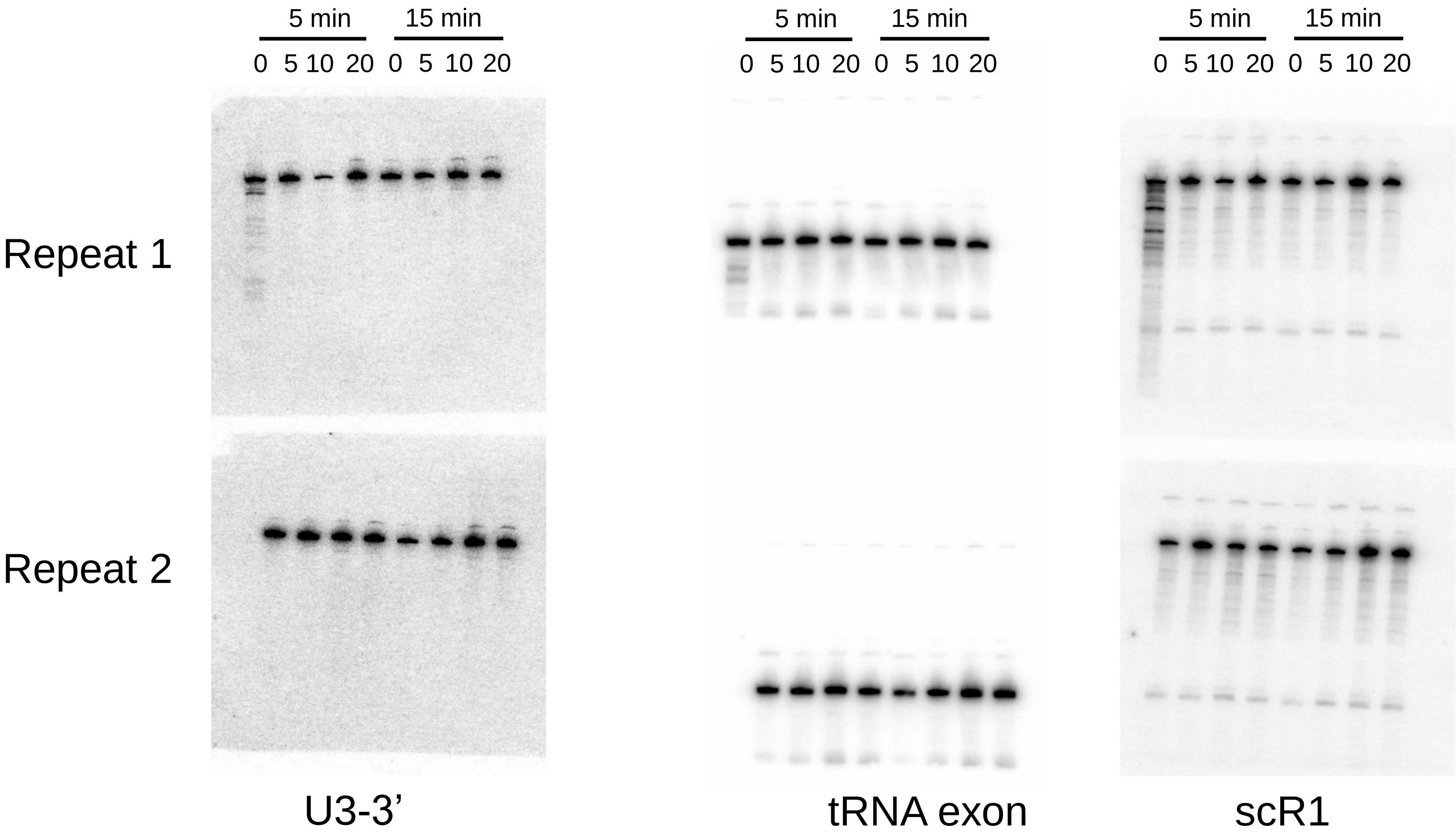

Original gel: Figure 2

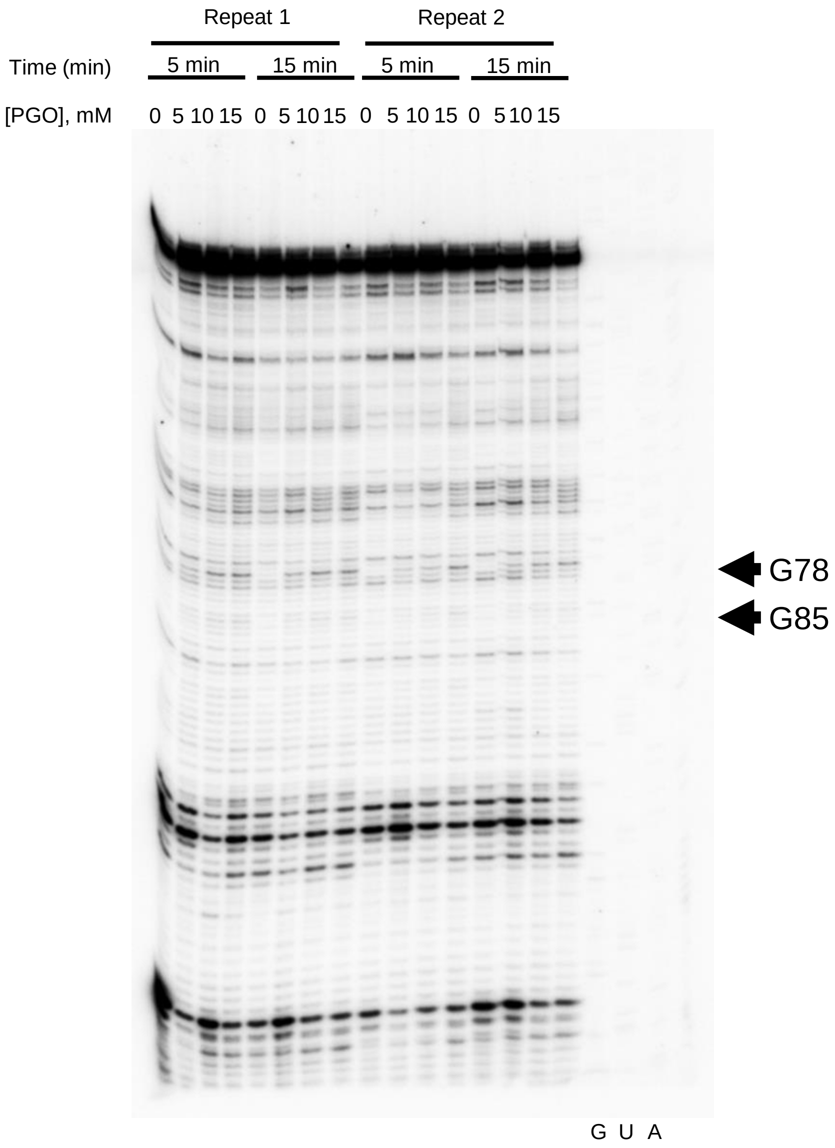

PGO

Original gel: Figure 4

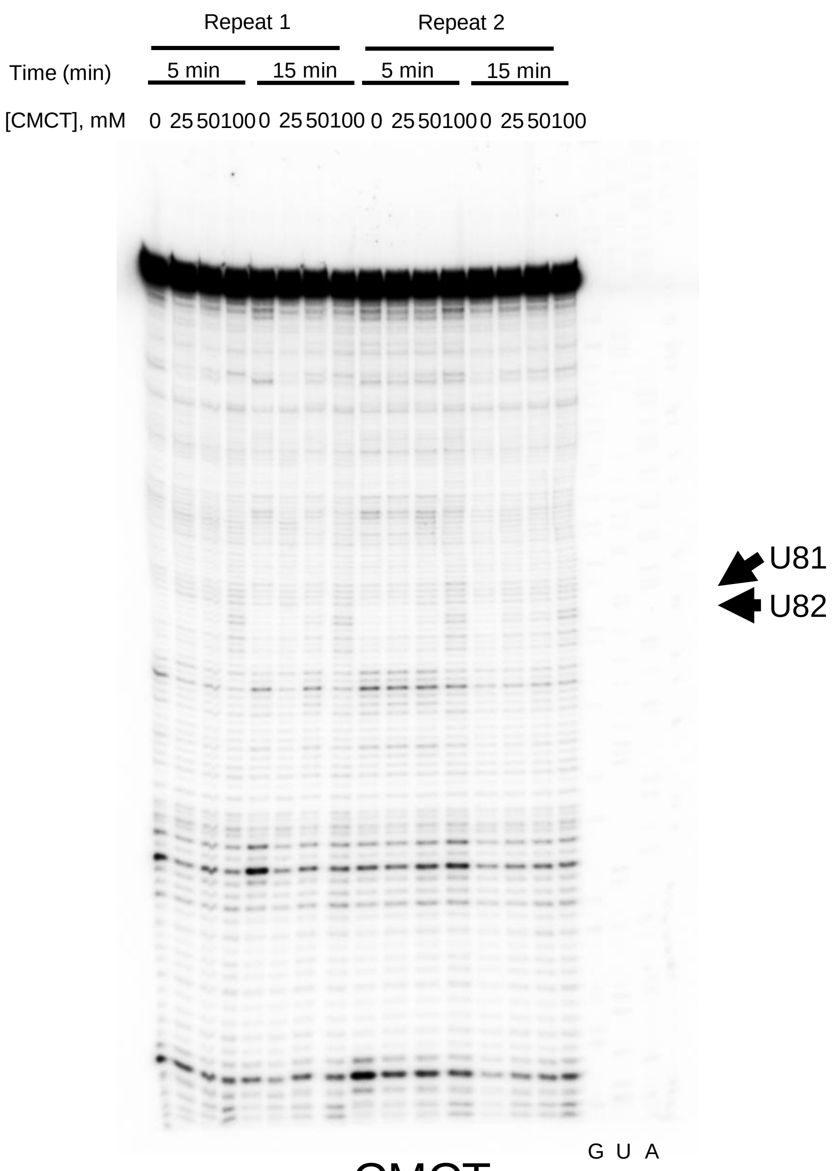

CMCT

Original gel: Figure 5

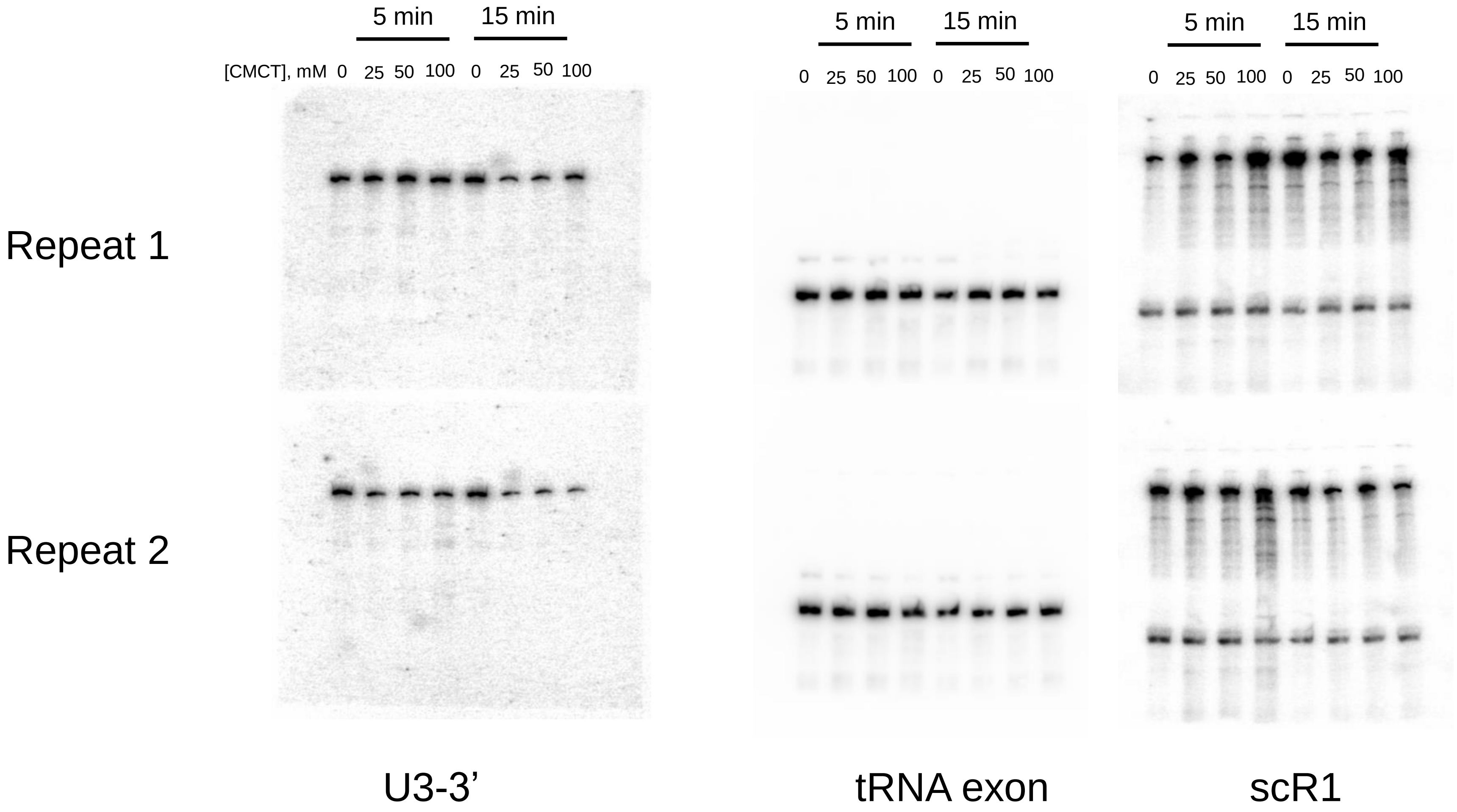

Original gel: Figure 6
